# Caregivers’ perceptions of fluorescence-guided surgery (FGS) in pediatric oncology: a questionnaire-based study

**DOI:** 10.1007/s00383-026-06463-x

**Published:** 2026-05-06

**Authors:** Irene Paraboschi, Ugo Maria Pierucci, Michela Marinaro, Cristina Di Franco, Eugenia Cao di San Marco, Elena Vegni Anna Maria, Gloria Pelizzo

**Affiliations:** 1 Department of Biomedical and Clinical Science, University of Milano, Via Giovanni Battista Grassi 74, 20157 Milan, Italy; 2 Department of Pediatric Surgery, ¨Vittore Buzzi¨ Children´s Hospital, Via Lodovico Castelvetro, 32, 20154 Milan, Italy; 3 Department of Mental Health and Dependencies, Unit of Clinical Psychology, Santi Paolo and Carlo Hospitals, Milan, Italy; 4 Department of Health Science, University of Milan, Milan, Italy

**Keywords:** Fluorescence-guided surgery, Pediatric surgical oncology, Caregivers, Perception, Surgical innovation

## Abstract

**Aim:**

To quantitatively assess parental engagement, perceptions, and decision-making attitudes toward fluorescence-guided surgery (FGS) in pediatric solid tumors.

**Methods:**

A structured questionnaire was administered to caregivers of children with Beckwith–Wiedemann syndrome in collaboration with the “Associazione Italiana Sindrome di Beckwith–Wiedemann”, facilitating outreach to families within the national support network. Emotional responses (optimism, hope, confidence, anxiety, worry, and uncertainty) and perceived support were assessed using 5-point Likert scales (1 = not at all, 5 = extremely). The survey also explored prior awareness of FGS, perceived impact on surgical outcomes and recurrence risk, openness to clinical trial enrollment, concerns, and information needs. Data were analyzed descriptively; open-ended responses were thematically coded.

**Results:**

Twenty-eight caregivers completed the survey. 86% of children had undergone tumor resection, predominantly for nephroblastoma (85%). Preoperative counseling was considered clear by 79% of caregivers, and perceived support from the medical team was high, with 68% reporting the maximum Likert score (5/5). Prior awareness of FGS was limited (11%); however, after explanation, acceptance was strong. Trust in surgical innovation was high (75% reporting maximum confidence), and 86% believed FGS could improve surgical safety and effectiveness. All respondents reported increased reassurance from the availability of technological support, and 71% perceived a potential reduction in recurrence risk. Emotional responses were predominantly positive, with high levels of confidence, hope, and optimism (75% maximum score), while anxiety, worry, and uncertainty were generally low. Qualitative analysis identified 3 main themes: strong trust and reassurance toward innovation, safety-related concerns focused on toxicity and long-term effects, and a clear need for transparent, detailed risk–benefit information prior to decision-making and potential clinical trial enrollment.

**Conclusions:**

Parents demonstrate high engagement and strong support for FGS, emphasizing the importance of transparent communication, shared decision-making, and active parent–patient involvement to facilitate clinical translation.

## Introduction

Fluorescence-guided surgery (FGS) is an evolving innovation in pediatric surgical oncology that enables real-time intraoperative visualization of tumor margins, vascular structures, lymph nodes, and occult residual disease through near-infrared (NIR) imaging [1–5].

By providing functional contrast beyond conventional white-light exposure, FGS has the potential to enhance surgical precision while minimizing morbidity. This is an especially important goal in children, where limited anatomical space and the need for long-term functional preservation demand maximal accuracy with minimal tissue sacrifice [1–5].

Early pediatric clinical experiences using indocyanine green (ICG) suggest that fluorescence imaging can improve intraoperative decision-making. In organ-preserving procedures such as nephron-sparing surgery for pediatric renal tumors, fluorescence guidance has been proposed to balance oncologic radicality with preservation of healthy renal parenchyma [6, 7]. In hepatoblastoma and pediatric liver metastases, NIR fluorescence has enabled identification of sub-centimeter and satellite lesions missed on preoperative imaging or palpation [8–10]. Similarly, in pulmonary metastasectomy, fluorescence imaging has revealed additional nodules compared with conventional techniques [11–13]. Beyond tumor detection, FGS may enhance lymph node mapping and staging accuracy [14–16] and assist vascular and perfusion assessment during complex resections [17]. Moreover, novel tumor-specific monoclonal antibodies targeting neuroblastoma-associated antigens, such as anti-GD2–based probes, are currently under preclinical and early translational investigation to enable more precise intraoperative identification of tumor margins [18–21].

Although long-term oncologic data remain limited and prospective validation is ongoing, FGS offers theoretical benefits including improved margin status, enhanced detection of occult disease, more accurate staging, and reduced unnecessary tissue sacrifice [1–5].

However, surgical innovation in children unfolds within a distinct ethical framework, as decisions are mediated by caregivers who must weigh potential benefits against uncertainty. While technical feasibility and early outcomes are increasingly reported, parental perception, trust in innovation, and acceptance of novel intraoperative technologies remain insufficiently explored. Understanding caregiver perspectives is therefore essential for the responsible and ethically sound integration of FSG techniques into pediatric oncologic practice.

The aim of this study was, therefore, to evaluate caregivers’ perceptions, emotional responses, and decision-making attitudes towards FGS in pediatric surgical oncology.

## Materials and methods

### Study design

A cross-sectional, questionnaire-based study was conducted between October 2025 and February 2026 to explore caregivers’ perceptions, emotional responses, and decision-making attitudes toward FGS in pediatric surgical oncology. The study was designed as an exploratory survey to assess parental engagement, perceived benefits and risks, trust in surgical innovation, and openness to potential clinical trial participation in the context of emerging intraoperative technologies.

### Study population and recruitment

Caregivers of children diagnosed with Beckwith–Wiedemann syndrome (BWS) were identified through collaboration with the “Associazione Italiana Sindrome di Beckwith–Wiedemann”. A total of 28 caregivers were contacted and invited to participate in the study. Eligibility criteria included being a parent or primary caregiver of a child with a confirmed diagnosis of BWS and having the ability to understand and complete the questionnaire in Italian. Participation was voluntary, responses were collected anonymously, and no financial compensation was provided. Caregivers were informed about FGS through dedicated information leaflets containing both conventional (white-light) and fluorescence images illustrating the aids of FGS in the surgical procedure.

### Questionnaire development

A structured questionnaire was specifically developed for this study and organized into four main domains: general clinical information, prior surgical experience, perceptions of FGS, and emotional and decision-making impact.

The first section collected demographic and clinical data.

The second section explored caregivers’ experience with surgical management, including clarity of preoperative counseling, communication regarding surgical margins, and perceived support from the medical team. Perceived support was assessed using a 5-point Likert scale ranging from 1 (not at all supported) to 5 (very supported).

The third section evaluated perceptions of FGS. Caregivers were asked about prior awareness of FGS, perceived impact of the technology on surgical safety and efficacy, perceived effect on recurrence risk, and overall reassurance associated with the availability of technological support during surgery. The presence of concerns was explored through both closed-ended and open-ended questions.

The fourth section assessed emotional responses to surgical innovation, including levels of confidence, hope, optimism, anxiety, worry, and uncertainty. These constructs were measured using 5-point Likert scales (1 = not at all; 5 = extremely).

Additional questions investigated preferred modalities for receiving information about innovative technologies, willingness to consider participation in a clinical trial, and the type of information required before making such a decision. Open-ended questions allowed respondents to elaborate on concerns, informational needs, and additional comments.

### Data collection and analysis

Completed questionnaires were collected and entered a dedicated database for analysis. No identifiable personal information was recorded. All procedures performed in this study involving human participants were conducted in accordance with the ethical standards of the institutional and/or national research committee and with the 1964 Helsinki Declaration and its later amendments or comparable ethical standards. According to our Institution’s regulations (i.e., Department of Pediatric Surgery, Buzzi Children’s Hospital, Milan, Italy), the need for ethics approval for this non-interventional study based on retrospectively obtained and anonymized data was waived. The confidentiality of the collected information was ensured in accordance with Regulation (EU) 2016/679 (GDPR) and Legislative Decree n.101/18. The survey was conducted anonymously, and no identifiable personal data were collected; participation was voluntary.

Quantitative data were analyzed descriptively and are presented as frequencies and percentages for categorical variables. Likert-scale responses were analyzed as ordinal variables and reported as distribution frequencies across response categories. Qualitative responses were analyzed using thematic analysis. Two independent reviewers examined open-ended responses and categorized them into recurrent thematic domains, including safety concerns, toxicity and chemical composition of fluorescent agents, technical reliability, risk–benefit evaluation, and informational needs. Given the exploratory nature of the study and the limited sample size, no inferential statistical testing was performed. Information regarding clinical variables and tumor resection margins was based on caregiver-reported data and was not independently verified against clinical records.

## Results

### Participant characteristics

**Fig. 1:**
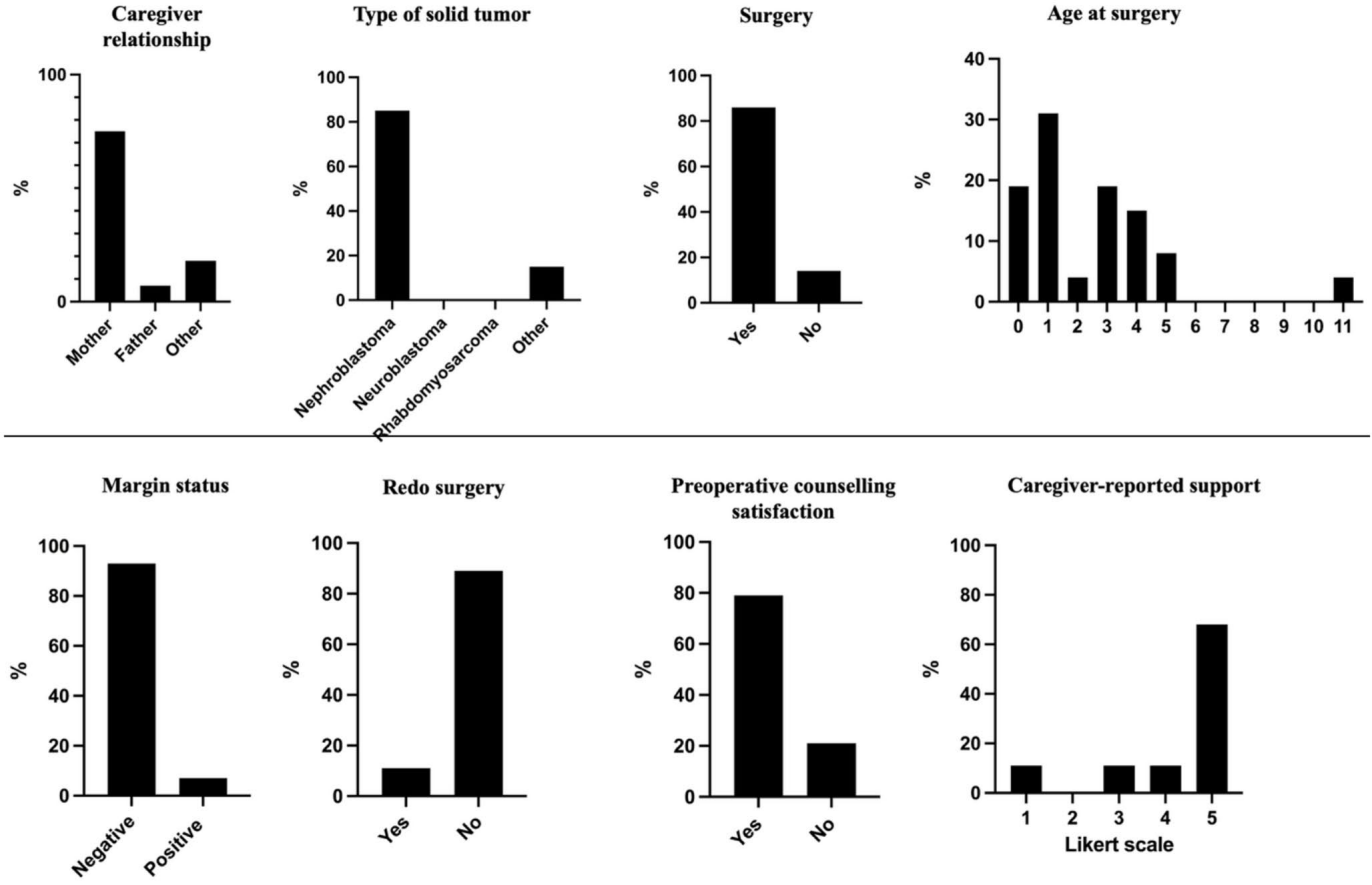
Demographic, clinical, and caregiver-reported characteristics of the study population

A total of 28 caregivers completed the questionnaire. Most respondents were mothers (*n* = 21, 75%), followed by fathers (*n* = 2, 7%) and other caregivers (*n* = 5, 18%). The ages of the children at the time the questionnaire was completed were widely distributed, encompassing a diverse pediatric and adolescent population. Age at surgical resection was median 1.5 (IQR 1–3.5) years, range 0–11 years. The most frequent age at surgery was 1 year (31%), followed by 3 years (20%) and infancy (20%), highlighting the early timing of oncologic interventions in this population. Nephroblastoma (Wilms tumor) represented the predominant diagnosis (85%), with a small number of caregivers reporting other tumor types (15%). Twenty-four children (86%) had undergone surgical resection. Complete tumor resection was reported in 82% of cases, whereas 7% of caregivers indicated incomplete resection and 11% were uncertain. Despite the high reported rate of complete resection, 7% of the caregivers reported having been informed of positive surgical margins postoperatively and a second surgical procedure was required. Redo surgery due to tumor recurrence was reported in 7% of cases (Fig. 1).

### Surgical experience and perceived support

Preoperative counseling was considered clear and satisfactory by 22 caregivers (79%), while 6 respondents (21%) reported insufficient clarity regarding surgical complexity. Perceived support from the medical team was consistently high. Nineteen caregivers (68%) selected the highest Likert score (5/5), and an additional 3 (11%) selected 4/5, indicating that nearly 4 out of 5 respondents felt strongly supported throughout the treatment pathway (Fig. 1).

### Awareness and perceptions of fluorescence-guided surgery

**Fig. 2:**
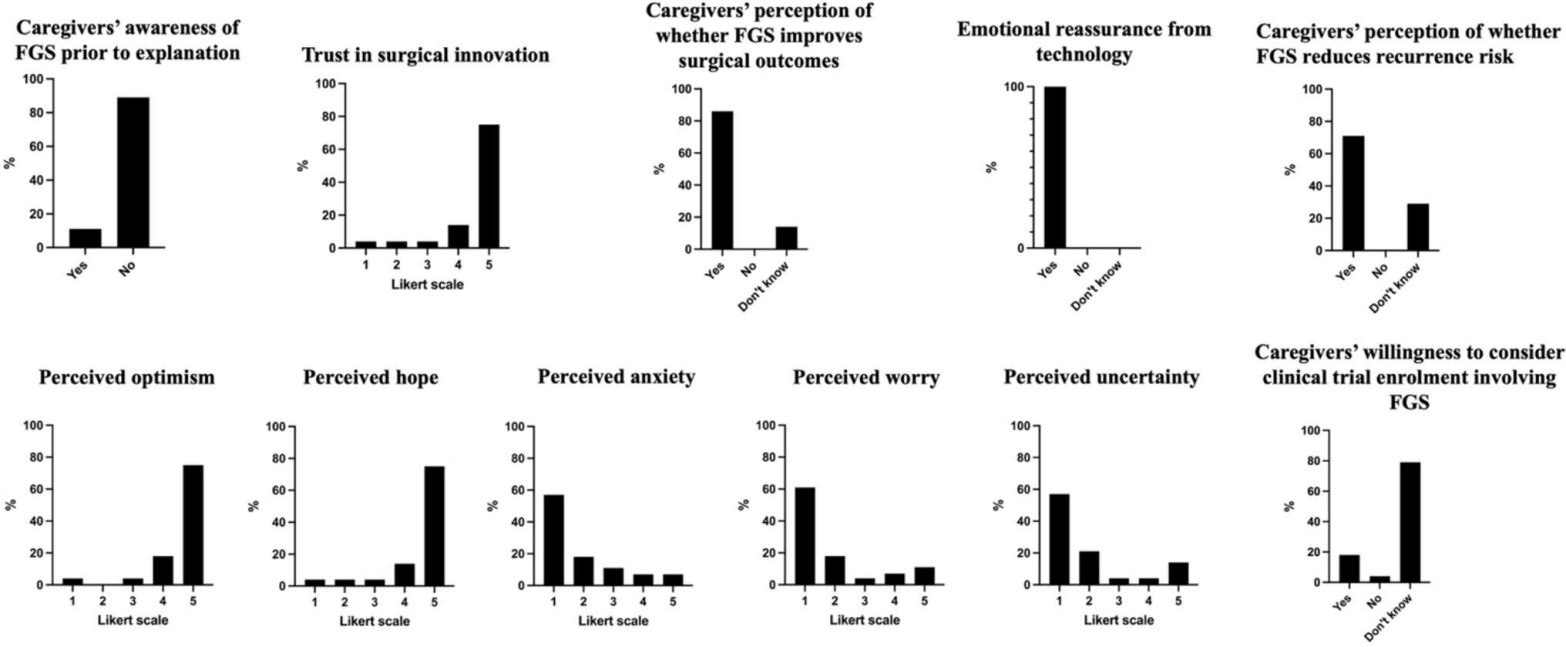
Caregiver perceptions and attitudes toward fluorescence-guided surgery (FGS)

Prior awareness of FGS was limited, with only 3 caregivers (11%) reporting previous knowledge of this technology. Despite this low baseline awareness (11%), acceptance of the technology was markedly high after explanation, with 86% of caregivers believing it could improve surgical safety and efficacy and 100% reporting increased reassurance. Trust in surgical innovation was similarly elevated: 21% (75%) expressed the highest possible confidence level (5/5), while only a small minority of caregivers (11%) reported low levels of trust (scores 0–1). Regarding recurrence, 20 caregivers (71%) believed that FGS could reduce recurrence risk and improve chances of cure, whereas 8 respondents (29%) expressed uncertainty. Notably, no respondent perceived the technology as potentially harmful in terms of oncologic outcome (Fig. 2).

### Emotional impact of surgical innovation

Emotional responses to the introduction of innovative surgical technologies were predominantly positive. High levels of confidence, hope, and optimism were consistently reported, with 75% of respondents selecting the maximum Likert score (5/5) for each of these constructs. In contrast, negative emotional responses were comparatively limited. Anxiety scores were predominantly low (0–2) in 75.0% of respondents and only 14% reported high levels of anxiety (scores 4–5). Similarly, low levels of worry (scores 0–2) were reported by 79% of caregivers, and low levels of uncertainty by 79%. Although higher uncertainty (scores 4–5) was reported by 18% of respondents, this did not appear to override the overall confidence in innovation (Fig. 2).

### Thematic analysis of open-ended responses

**Fig. 3:**
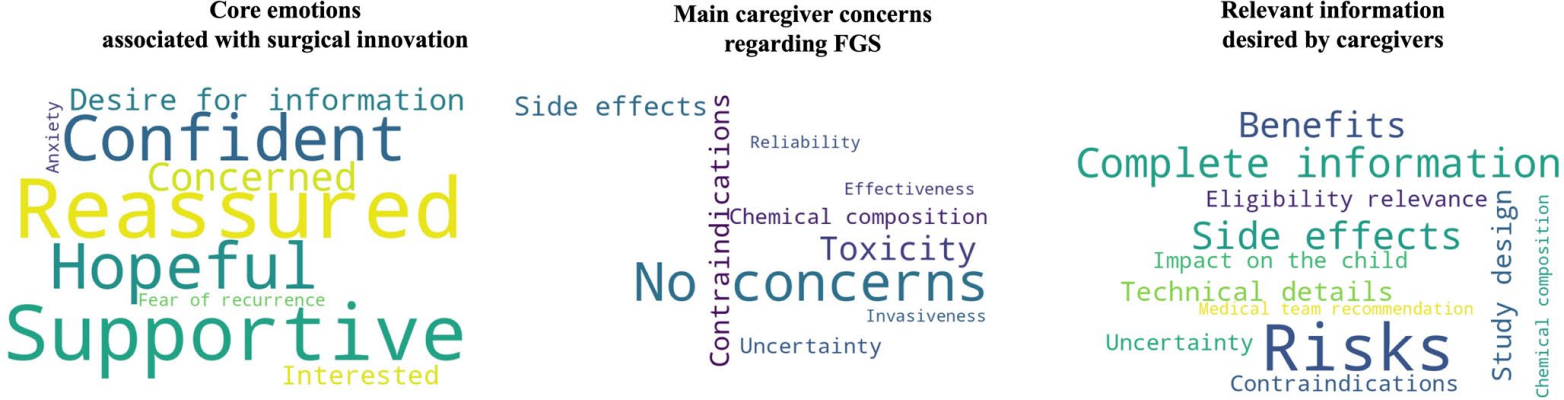
Word clouds illustrating caregivers’ qualitative responses. The three panels summarize (left) core emotions expressed toward fluorescence-guided surgery (FGS), (center) main concerns related to the technology, and (right) information needs prior to decision-making

Qualitative thematic analysis identified three principal domains. First, positive core emotions emerged strongly, including reassurance, supportiveness, hope, and trust in medical teams. Many caregivers expressed explicit confidence in surgical expertise and openness toward innovation when framed as safe and evidence based. Second, concerns centered primarily on safety. Recurrent themes included toxicity of fluorescent agents, potential short- and long-term side effects, chemical composition, elimination kinetics, and reliability of emerging technology. These concerns were framed not as opposition but as requests for clarification. Third, information priorities consistently emphasized the need for complete, transparent, and technically detailed communication. Caregivers requested balanced explanations of risks and benefits, clarity regarding eligibility criteria, and reassurance regarding clinical trial oversight and safety monitoring. Overall, the data reveal a pattern of high engagement, strong trust in medical teams, positive emotional alignment with innovation, and a desire for transparent risk communication rather than resistance to technological advancement (Fig. 3).

## Discussion

FGS is increasingly emerging as a promising adjunct in pediatric surgical oncology [1–5]. Although still in a translational phase for many tumor types, the integration of NIR imaging and tumor-targeted probes has the potential to enhance intraoperative precision, optimize margin status, and reduce unnecessary tissue sacrifice [1–5].

In pediatric patients, where anatomical constraints, long-term functional preservation, and oncologic radicality must be carefully balanced, such technological innovation carries relevance [1–5]. Early pediatric experiences with ICG and tumor-targeted agents have demonstrated feasibility and encouraging intraoperative utility, yet long-term oncologic outcome data remain limited [1–5].

Within this evolving landscape, understanding not only technical performance but also caregiver perspectives become essential.

Our study provides preliminary evidence that caregivers of children with Beckwith–Wiedemann syndrome, a genetic overgrowth disorder associated with a significantly increased risk of developing pediatric solid tumors, particularly Wilms tumor and hepatoblastoma, exhibit a generally positive and receptive attitude toward the concept of FGS.

Despite limited prior awareness of the technology, once explained, the majority perceived FGS as potentially beneficial for improving surgical safety and reducing recurrence risk. Importantly, reassurance derived from the availability of additional technological support, such as fluorescence imaging systems enhancing tumor visualization, was universal. These findings suggest that parental acceptance of intraoperative innovation may be high when clear explanations are provided and when innovation is framed as an adjunct to, not a replacement for, surgical expertise.

Notably, our results also highlight a central theme: trust in the medical team appears to mediate acceptance of innovation. High levels of perceived support and confidence in surgical innovation were consistently reported. This aligns with prior qualitative work demonstrating that pediatric surgeons favor collaborative educational approaches that engage parents in decision-making discussions, particularly in high-stakes contexts such as oncology [22]. In that study, surgeons emphasized the importance of “reading the room” and tailoring communication to parental emotional needs, principles that appear directly relevant when introducing novel technologies such as FGS.

The growing emphasis on patient and public involvement (PPI) in pediatric research provides further context for interpreting our findings. PPI is increasingly recognized as a mechanism to enhance research relevance, ethical accountability, recruitment, and translational impact [23].

In pediatric settings, engagement must be developmentally appropriate and family-centered, acknowledging the triadic relationship between child, caregiver, and clinician. Our study contributes to this literature by extending the PPI framework into the domain of surgical technological innovation, an area where caregiver perspectives have been insufficiently explored.

Recent systematic work has demonstrated that most pediatric engagement initiatives remain at the level of consultation rather than true partnership or co-leadership [24].

Similarly, reviews of pediatric patient engagement in clinical care and research show increasing activity but ongoing heterogeneity in methods and reporting [25].

Our findings reflect this broader trend: caregivers were primarily consulted regarding their perceptions of FGS, rather than actively involved in shaping its implementation pathways. Nonetheless, even consultative engagement provides valuable insight into safety concerns, informational needs, and willingness to consider clinical trial participation.

Importantly, concerns expressed by caregivers in our study were not directed at the concept of innovation per se, but rather at issues of toxicity, long-term safety, chemical composition of fluorescent agents, and study design transparency. These concerns echo the ethical imperative articulated in PPI literature that meaningful engagement requires not only information provision but also reciprocal dialogue regarding risks and uncertainties [26]. Caregivers in our cohort requested detailed, balanced explanations of risks and benefits before considering clinical trial enrollment—highlighting that acceptance of innovation is conditional upon transparent communication.

Our findings also align with broader models of patient engagement within enhanced recovery and surgical pathways. Patient activation and structured perioperative counseling have been shown to improve satisfaction and outcomes in pediatric surgical populations [26].

Although FGS represents a distinct technological intervention, its integration into clinical practice will likely require similar educational frameworks that define expectations, clarify rationale, and reinforce collaborative partnership.

From an ethical perspective, pediatric surgical innovation occupies a uniquely sensitive space. Decisions are mediated by caregivers, emotional burden is high, and uncertainty may be amplified when novel intraoperative tools are introduced. However, our data suggest that caregivers do not inherently resist innovation; rather, they seek clarity, reassurance, and safety validation. This distinction is crucial for translational implementation. Resistance to innovation may be less about novelty and more about communication quality and perceived transparency.

Therefore, the integration of FGS into pediatric oncologic surgery should be accompanied by structured caregiver education, explicit discussion of safety data, and opportunities for bidirectional dialogue. Information delivery strategies should combine direct physician communication with supportive educational materials, as preferred by most respondents. Moreover, engagement strategies should move beyond simple consent processes toward structured involvement models consistent with established PPI frameworks.

From a clinical perspective, these findings have direct implications for future practice. They underscore the importance of delivering clear, structured, and transparent information when introducing innovative surgical technologies, such as FGS. Particular attention should be given to addressing caregiver concerns related to safety, potential toxicity, and long-term effects, which emerged as key themes in this study. The use of standardized communication strategies, including visual aids and tailored informational materials, may improve understanding and trust. Incorporating caregiver perspectives into the clinical pathway may ultimately support shared decision-making and facilitate the safe implementation of emerging technologies in pediatric surgical oncology.

This study has several limitations. First, the relatively small sample size and the focus on a specific caregiver population (i.e., families of children with Beckwith–Wiedemann syndrome) may limit the generalizability of the findings to other pediatric oncology settings. Second, as this was a cross-sectional survey-based study, responses reflect perceptions at a single time point and may be influenced by individual experiences and context. Third, although emotional responses were quantified using Likert scales, a more in-depth qualitative approach could provide a deeper understanding of caregiver decision-making processes. In addition, caregiver-reported clinical information, including tumor resection margins, may be subject to recall bias and was not independently verified against medical records. Finally, as none of the patients had direct experience with FGS, responses were based on hypothetical acceptance following explanation of the technology, which may differ from attitudes in real clinical implementation.

## Conclusion

As FGS advances from technical feasibility toward broader clinical adoption in pediatric oncology, integrating caregiver perspectives into translational pathways becomes imperative. Our findings suggest that caregivers are largely supportive of innovation when adequately informed and when trust in the surgical team is strong. However, safety transparency, risk–benefit clarity, and structured engagement strategies are essential to ethically and sustainably embed FGS into pediatric surgical practice.

## Data Availability

No datasets were generated or analysed during the current study.
